# Selective decontamination and antibiotic resistance in ICUs

**DOI:** 10.1186/s13054-015-0967-9

**Published:** 2015-06-24

**Authors:** Nienke L. Plantinga, Marc JM Bonten

**Affiliations:** University Medical Center Utrecht, P.O. Box 85500, 3508 GA Utrecht, The Netherlands

## Abstract

Selective digestive decontamination (SDD) and selective oropharyngeal decontamination (SOD) have been associated with reduced mortality and lower ICU-acquired bacteremia and ventilator-associated pneumonia rates in areas with low levels of antibiotic resistance. However, the effect of selective decontamination (SDD/SOD) in areas where multidrug-resistant Gram-negative bacteria are endemic is less clear. It will be important to determine whether SDD/SOD improves patient outcome in such settings and how these measures affect the epidemiology of multidrug-resistant Gram-negative bacteria. Here we review the current evidence on the effects of SDD/SOD on antibiotic resistance development in individual ICU patients as well as the effect on ICU ecology, the latter including both ICU-level antibiotic resistance and antibiotic resistance development during long-term use of SDD/SOD.

## Introduction

Selective digestive decontamination (SDD) refers to the prophylactic treatment of selected patients with an oropharyngeal paste and enteral suspension containing antimicrobials (usually tobramycin, colistin and amphotericin B) as well as an intravenous antibiotic during the first 4 days of ICU admission (usually a second-generation cephalosporin). The purpose of the treatment is to eradicate potential pathogenic microorganisms from the oropharynx and digestive tract of patients at risk for nosocomial infections (ventilated patients, neutropenic patients and neonates). The targeted potential pathogenic microorganisms include aerobic Gram-negative bacteria (GNB), methicillin-susceptible *Staphylococcus aureus* and yeasts, and once a patient has been successfully decolonized the unaffected anaerobic flora would offer prevention against new colonization with potential pathogenic microorganisms, a principle called colonization resistance [1]. Selective oropharyngeal decontamination (SOD) consists of SDD without the enteral suspension and without intravenous antibiotics.

Thirty years of selective decontamination (SDD/SOD) studies include >50 randomized studies and >10 meta-analyses. In the most recent meta-analysis, pooled analyses of the available evidence suggest that SDD and SOD are associated with improved survival [2]. Yet the amount of heterogeneity between studies owing to differences in study designs, included patient populations, interventions and diagnostic procedures justifies caution in data interpretation. Moreover, beneficial effects reported from more meta-analyses should not be seen as cumulative evidence, because all analyses rely on the same data. Notwithstanding these beneficial effects, the risks associated with the prophylactic use of antibiotics must also be considered. This debate is fed by arguments concerning patient safety and ecological safety in an era of increasing antibiotic resistance. Arguments against widespread use of SDD/SOD are supported by reported outbreaks of antibiotic-resistant bacteria during the use of SDD, development of secondary resistance within patients exposed to SDD, and failing attempts to control outbreaks by implementation of SDD. Arguments pro SDD/SOD are supported by reports and a recent meta-analysis about the absence of resistance development during its use [3], and descriptions of outbreaks that were actually constrained by SDD.

This nonsystematic review first aims to provide an overview of the available data on the relationship between use of SDD/SOD and antibiotic resistance in individual patients admitted to ICUs with low levels of antibiotic resistance. Although this will hamper extrapolation of findings to other settings, the review does reflect a real-life research setting in which an important reason for heterogeneity between units – that is, antibiotic resistance epidemiology – is prevented. The insights derived in these settings can serve to design studies in settings with higher levels of antibiotic resistance. Since a systemic review and meta-analysis was performed in 2013 [3], this description is limited to a selection of studies that – in our opinion – best answer the question(s) addressed.

Secondly, we will review the effects of SDD/SOD on the occurrence of ICU-level antibiotic resistance and on antibiotic resistance development during long-term use. These ecologic studies have mainly been performed in ICUs with low levels of antibiotic resistance.

For completeness, findings of universal and targeted use of SDD/SOD and antibiotic resistance in ICUs where multidrug-resistant Gram-negative bacteria (MDR-GNB) were endemic are summarized in table form. Finally, three randomized controlled trials (RCTs) with SDD applied specifically to patients colonized with MDR-GNB will be discussed.

## Selective decontamination and antibiotic resistance in individual patients

In this nonsystematic review we describe the effects of SDD/SOD on antibiotic resistance in individual patients admitted to ICUs with low levels of antibiotic resistance, as obtained from studies: that applied SDD/SOD in all eligible patients (that is, not in specific subgroups only); in ICUs with absence of endemicity with methicillin-resistant *Staphylococcus aureus* (MRSA), vancomycin-resistant enterococci (VRE) or carbapenem-resistant Enterobacteriaceae (CRE) and less than 10 % of Gram-negative infections caused by extended-spectrum beta-lactamases (ESBL); in which there was some form of random treatment allocation, with reported data on the occurrence of antibiotic-resistant GNB; and that were published since 2000.

Most studies investigating selective decontamination used individual patient randomization, creating a mixture of patients receiving and not receiving SDD/SOD in the ICU. In 2002 such an individual RCT randomizing SDD (topical polymyxin and gentamicin, 4-day course of ciprofloxacin intravenously) described no ‘remarkable differences between the groups with respect to the isolation of resistant bacteria’ from surveillance cultures [4]. When comparing SDD with placebo, 5/265 versus 7/262 patients developed infections with GNB resistant to ciprofloxacin, 4/265 versus 10/22 patients developed infections with GNB resistant to gentamicin and 2/265 versus 18/262 patients developed infections with GNB resistant to polymyxin.

de Jonge and colleagues were among the first to determine the effects of SDD when applied to all patients in one unit, who were compared with patients treated in a similar unit in which SDD was not used [5]. This clustered approach is optimal to quantify the effects of interventions in which patient dependency cannot be excluded, such as measures that prevent colonization and infection by modulation of the unit-wide bacterial ecology, or interventions that should reduce cross-transmission, such as hand hygiene. During 24 months patients were admitted to each unit based on the availability of beds, and were randomized if beds were available in both units. In this study, SDD was associated with higher levels of antibiotic susceptibility of GNB to ceftazidime, ciprofloxacin, imipenem and tobramycin. VRE were isolated in four and five patients from the SDD unit and the control unit, respectively, and MRSA was not detected. Moreover, SDD was associated with a 35 % reduction in ICU mortality.

In a French ICU, SDD was compared with chlorhexidine body washing plus intranasal mupirocin and with placebo in a 2 × 2 factorial design [6]. Proportions of patients developing infections with colistin-resistant GNB ranged from 11 % in patients receiving double placebo treatments to 2 % among patients receiving both SDD and chlorhexidine body washing/mupirocin (*P* = 0.005). A similar (although nonsignificant) trend was observed for tobramycin-resistant GNB infections, ranging from 17 % to 9 % in double placebo-treated patients and SDD plus chlorhexidine/mupirocin-treated patients, respectively.

Detailed information on antibiotic resistance is also available from a multicenter cluster-randomized crossover trial in 13 Dutch ICUs, in which (in random order) SDD, SOD and standard care (that is, no SDD or SOD) were compared during periods of 6 months per intervention [7]. Almost 90 % of all patients that stayed in the ICU for at least 48 hours were included. In this study, ICU-acquired bacteremia with highly resistant microorganisms (HRMO; mainly GNB) occurred less frequently during SDD, as compared with SOD and standard care (crude odds ratios (95 % confidence intervals): SDD vs. SOD, 0.37 (0.16 to 0.85); SDD vs. standard care, 0.41 (0.18 to 0.94); SOD vs. standard care, 1.10 (0.59 to 2.07)) [8]. Furthermore, both SDD and SOD were associated with less acquisition of respiratory tract colonization with most relevant HRMO, with crude odds ratios (95 % confidence intervals) of 0.58 (0.43 to 0.78) for SDD and 0.65 (0.49 to 0.87) for SOD as compared with standard care [8]. Acquired respiratory tract colonization with Enterobacteriaceae resistant to cefotaxime, to tobramycin or intrinsically resistant to colistin – which are part of SDD – occurred less frequently in patients receiving SDD, as compared with those receiving standard care or SOD [8]. Tobramycin resistance in glucose nonfermenting GNB was highest during SDD [8].

Subsequent analyses on resistance development against colistin revealed that acquisition rates of carriage with colistin-resistant GNB were 0.8, 1.1 and 0.7 per 1,000 patient-days at risk during standard care, SOD and SDD, respectively [9]. Conversion rates of colistin-susceptible GNB to colistin-resistant strains were 0.5, 0.5 and 0.7 per 1,000 patient-days at risk. Yet these events occurred (by definition) only in patients colonized with GNB, and therefore conversion rates among colonized patients were highest during SDD (1.1, 2.6 and 3.6 during standard care, SOD and SDD, respectively). Moreover, conversion to colistin resistance occurred preferentially in GNB already resistant to tobramycin. It was concluded that, in Dutch ICUs, the prevalence of colistin resistance was low and that resistance development occurred infrequently and was not associated with SDD/SOD, but that the presence of tobramycin resistance increases the risk of secondary colistin resistance.

In another analysis, the effect of SDD on intestinal decolonization was compared for patients with intestinal colonization with Enterobacteriaceae that were either susceptible or resistant to cephalosporins or aminoglycosides at the time of ICU admission [10]. Intestinal decolonization rates were comparable for Enterobacteriaceae susceptible to and resistant to cephalosporins (343/430 (80 %) vs. 56/77 (73 %) respectively, *P* = 0.17). However, in aminoglycoside-resistant Enterobacteriaceae, SDD was less successful in eradication (368/457 (81 %) vs. 31/50 (62 %) for Enterobacteriaceae susceptible to versus resistant to aminoglycosides, respectively; *P* < 0.01).

In the largest, and most recent, multicenter cluster-randomized crossover study, SDD was compared with SOD in 16 Dutch ICUs [11]. In this pragmatic study of almost 12,000 patients, all patients with an ICU length of stay >48 hours and all patients having received one dose of SOD or SDD (that is, all patients with an expected ICU length of stay >48 hours) were the eligible study population (*N* = 11,997), thereby avoiding selection bias. Cumulative incidences of ICU-acquired bacteremia during SOD and SDD were 5.9 % and 4.6 %, respectively (*P* = 0.002), and were 0.6 % and 0.4 % for episodes caused by HRMO during SOD and SDD, respectively (*P* = 0.27).

In conclusion, the results from these large SDD/SOD trials in settings with low levels of antibiotic resistance strongly suggest that SDD and SOD can be safely used in the treatment of ICU patients. Microbiological surveillance, especially with regard to aminoglycoside and colistin susceptibility, is recommended to monitor development of antibiotic resistance.

## Ecological effects of selective decontamination

The effect of SDD/SOD on antibiotic resistance is not only relevant for patients treated with these antimicrobials, but also for other and even future ICU patients. There are two important questions to be answered concerning the ecological effects of SDD/SOD.

First, what is the effect of SDD/SOD on ICU-level bacterial ecology, more specifically on the prevalence of antibiotic-resistant bacteria, in all patients? To answer this question, we searched for studies that assessed antibiotic resistance on an ICU level, either through regular point-prevalence sampling of all patients present in the ICU or by evaluation of routine cultures from all admitted patients (rather than from those treated with SDD/SOD only), and that had a control group, separated from the intervention in time or location, for comparison.

Second, does the long-term use of SDD/SOD change the prevalence of these bacteria? To answer this question we searched for studies that had collected resistance data during at least 3 years of SDD/SOD use, and in which antibiotic resistance was assessed either by time-trend analysis or by a comparison with an appropriate control group.

Again, almost all studies addressing these questions have been performed in settings with low levels of antibiotic resistance.

### ICU-level antibiotic resistance (short-term use of selective decontamination)

In two Dutch cluster-randomized multicenter studies with crossover, the ecological effects of SDD and SOD on antibiotic resistance were monitored prospectively with monthly point-prevalence surveys. These surveys included all patients present in the ICU at a certain time point, including those not receiving SDD or SOD. In the first study – investigating standard care, SOD and SDD in randomized order in 13 ICUs – completeness of rectal and respiratory samples was 87 % and 82 %, respectively [7]. Antibiotic resistance amongst *Escherichia coli*, *Klebsiella pneumoniae*, *Pseudomonas aeruginosa* and *Enterobacter cloacae* was lowest during SDD (as compared with standard care and SOD) for all 16 pathogen–antibiotic combinations as well as for multidrug resistance. In a *post hoc* analysis of these data, the unit-wide prevalence of GNB resistant to ceftazidime, tobramycin or ciprofloxacin in rectal swabs was lowest during SDD, but the prevalence was higher in the months thereafter, suggesting a rebound effect [12]. In the second Dutch cluster-randomized study, 16 ICUs were randomized to 12 months of SDD and 12 months of SOD, in random order [11]. The unit-wide prevalence of antibiotic-resistant microorganisms was again measured with monthly point-prevalence surveys including 3,776 rectal specimens and 3,651 respiratory samples. The prevalence of respiratory tract colonization with HRMO was similar in both groups, but during SDD the prevalence of rectal colonization with such bacteria was lower (7.3 % during SDD vs. 12.7 % during SOD, *P* = 0.008). Yet the prevalence of aminoglycoside-resistant GNB in rectal swabs increased more pronouncedly during SDD (7 % per month vs. 4 % during SOD, *P* <0.05). The previously observed rebound effect of ceftazidime resistance after discontinuation of SDD was not confirmed in this study [12].

ICU-level effects of SDD/SOD on the prevalence of antibiotic-resistant GNB can also be measured through analyzing microbiological samples from all ICU patients (rather than from only patients receiving SDD/SOD or through point-prevalence surveys – the latter method precludes measurement of antibiotic resistance in all SDD/SOD-treated patients). This method was used in a single-center before–after study in France [13]. Here, implementation of SDD (without systemic antibiotics) was followed by a unit-wide reduction in the proportion of patients with an ICU-acquired infection caused by MDR-GNB; from 2.6 % 1 year before to 0.9 % during the first year of SDD (*P* = 0.003).

Whether 6 to 12 months of SDD/SOD use exerts sufficient antibiotic pressure to significantly increase antibiotic resistance in settings where it is rare is uncertain. The effects of long-term use of SDD/SOD will therefore be described below.

### Antibiotic resistance during long-term use of selective decontamination

The effects of long-term use of SDD/SOD (at least 3 years) have been addressed in two multicenter studies and two single-center studies. Two studies investigated antibiotic resistance in specific isolates from all ICU patients [14, 15], one study determined acquisition of antibiotic-resistant GNB in SDD-treated patients using surveillance and clinical microbiology results [16], and the most recent study used point-prevalence samples [17].

In a German study, antibiotic resistance (MRSA, VRE, tobramycin-resistant GNB) was evaluated during 5 years of SDD in a single unit, and this was compared with findings from 33 ICUs not using SDD [15]. Incidence rates of MRSA and tobramycin-resistant *P. aeruginosa* were lower in the ICU where SDD was used as compared with the pooled data of the other ICUs, and the opposite was observed for VRE, tobramycin-resistant *E. coli* and *K. pneumoniae.* The increase in VRE was explained by a hospital-wide outbreak and most episodes of carriage with tobramycin-resistant Enterobacteriaceae were not considered ICU acquired. The authors therefore concluded that in their setting with low baseline resistance levels, with a national surveillance program for resistance monitoring and with an active screen-and-isolate protocol for MRSA, SDD was safe during this period of time.

In a recent study, microbiological culture results from respiratory samples from patients in 38 Dutch ICUs over a period of 51 months were retrospectively analyzed [14]. In 17 ICUs SDD or SOD had been used continuously, in 13 ICUs SDD/SOD had not been used and in eight ICUs SDD or SOD was introduced during the period of data collection. Time-trend analyses did not reveal statistically significant increases in the occurrence of antibiotic-resistant GNB in ICUs continuously using SDD or SOD, while resistance increased for some pathogen–antibiotic combinations in ICUs not using SDD or SOD. In those ICUs in which SDD or SOD was introduced, there was an increase in colistin-resistant Enterobacteriaceae before introduction, followed by a reduction in resistance after its implementation.

In a Spanish ICU where SDD was the standard of care (with addition of vancomycin for oxacillin-resistant *S. aureus* carriers), both the prevalence of colonization with antibiotic-resistant GNB and/or oxacillin-resistant *S. aureus* on ICU admission as well as the incidence density of acquisition (both colonization and infection) with these bacteria during ICU admission were stable during 5 years [16].

In a *post hoc* analysis from two Dutch multicenter SDD/SOD studies [7,11], the unit-wide point prevalence of colistin-resistant GNB and of tobramycin-resistant GNB was compared during both study periods in five hospitals that continued to use SDD between studies. The average duration of uninterrupted SDD use between studies was 3.8 years and the average duration of uninterrupted SDD/SOD use including the study periods was 6.4 years (range 5.6 to 7.4 years). For both SDD and SOD there were nonsignificant reductions in resistance to both colistin and tobramycin in both respiratory and rectal samples [17].

In summary, in these ecological studies SDD/SOD appeared ecologically safe during longer periods of time (3 to 6.4 years) in ICUs with relatively low levels of antibiotic resistance. To the best of our knowledge there are no studies, using similar methodology, with different results. An international multicenter study on the effects of SDD and SOD on ICU-level ecology in countries with higher levels of antibiotic resistance is currently ongoing (ClinicalTrials.gov: NCT02208154).

Nevertheless, even in countries with low levels of antibiotic resistance, such as the Netherlands, outbreaks with resistant bacteria can occur while using SDD. In an attempt to control an ongoing outbreak caused by ESBL-producing and tobramycin-resistant *K. pneumoniae*, SDD was implemented and the prevalence of colistin resistance in these isolates increased from complete absence before introduction to 70 % (74 of 106 isolates) after introduction of SDD [18]. Almost all isolates (71 of 74) belonged to one specific clone. These findings illustrate that SDD should not be used to control transmission of MDR-GNB if classical control measures have failed.

## Selective decontamination in ICUs with high levels of antibiotic resistance

The effects of selective decontamination on antibiotic resistance are less well studied in settings with high levels of antibiotic resistance. We are aware of four observational studies and one small RCT (Table 1) that have been performed in ICUs where MDR-GNB were endemic (endemicity/outbreak of a certain species of MDR-GNB as described by the authors), all using SDD, applied either as universal treatment (*n* = 3) or as targeted treatment for identified carriers (*n* = 3). Most of these studies investigated the effects of SDD on elimination or persistence of carriage with resistant strains, and ecologic outcomes were not reported. Settings, study designs, methods (random treatment allocation was performed in one study only) and results differ extensively, precluding a clear interpretation.

**Table 1:** Effects of selective decontamination in ICUs where multidrug-resistant Gram-negative bacteria were endemic

Author, year, country	Design (duration), setting	MDR species; population	Intervention – targeted/universal; study groups	Treatment efficacy	Resistance to SDD	Reported conclusion
Brun-Buisson and colleagues, 1989, France [22]	Prospective observational study (10 weeks) followed by RCT (8 weeks), medical ICU	MDR-E; ICU LOS >2 days and admission severity of illness score >2 (*n* = 210)	Universal: SDD = neomycin, polymyxin E and nalidic acid (no IV); (1) no SDD (observation, *n* = 124), (2) no SDD (RCT, *n* = 50), (3) SDD (RCT, *n* = 36)	ICU-acquired colonization with MDR-E: 19.6 % vs. 10 % vs. 2.9 % (*P* < 0.01 for 1 vs. 3); infection/colonization clinical site with MDR-E: 9 % vs. 6 % vs. 0 %	Rectal colonization with species resistant to SDD: 32 % vs. 58 % (*P* = 0.02, 2 vs. 3)	‘… intestinal decontamination can be helpful to control outbreaks of multiresistant gram-negative bacilli, especially due to *Klebsiella* species, in the intensive care setting.’
Taylor and Oppenheim, 1991, USA [23]	Before–after study (2 + 2 months), multidisciplinary ICU	ESBL-KA; ICU LOS >48 hours (*n* = 33)	Universal: SDD = colistin, tobramycin and amphotericin (no IV); (1) no SDD (2 months, *n* = 15), (2) SDD (2 months, *n* = 18)	Colonization/infection ESBL-KA: 26.7 % vs. 0%	‘No gram-negative aerobes resistant to the SDD drugs or ceftazidime emerged during the SDD regimen’	‘SDD appears to be a useful tool for eradicating outbreaks due to Gram-negative aerobic bacilli.’
Decré and colleagues, 1998, France [24]	Observational study (12 months), infectious disease ICU	ESBL-KP; (1) all patients admitted to ICU, (2) colonized and/or infected patients (*n* = 404)	Universal vs. targeted: SDD = erythromycin and polymyxin E (no IV); (1) universal SDD (7 months, *n* = 239), (2) targeted SDD (5 months, *n* = 165)	ICU-acquired colonization/infection with ESBL-K: 10.0 % vs. 9.1 %; ICU-acquired infection with ESBL-KP: 7.5 % vs. 3.6 %	Not reported	’… prophylactic SDD failed to significantly reduce the incidence of acquisition of ESBL-producing strains.’
Agusti and colleagues, 2002, Spain [25]	Before–after study (2 + 2 months with 5 months between), ICU	*Acinetobacter baumannii*; patients with *A. baumannii* intestinal carriage, expected ICU LOS >5 days (*n* = 54)	Targeted: SDD = polymyxin E and tobramycin (no IV); (1) no SDD (*n* = 33), (2) SDD (*n* = 21)	*A. baumanni* colonization at ICU discharge: pharyngeal 78 % vs. 38 % (*P* = 0.03), rectal 91 % vs. 48 % (*P* < 0.001); *A. baumanni* in clinical samples: 81% vs. 45.5 % (*P* = 0.05)	‘No resistance to colistin developed during the study’	‘… SDD may be beneficial, decreasing the intestinal reservoir in ICU patients with *A. baumannii* colonization […]. … if SDD is used in the setting of an *A. baumannii* outbreak, decontamination should not be restricted to the digestive tract, but applied also to the skin …’
Lubbert and colleagues, 2013, Germany [26]	Retrospective cohort (28 months), surgical ICU	KPC-2-KP; patients colonized/infected with KPC-2-KP (*n* = 90)	Targeted: SDD = colistin and gentamicin (no IV); (1) SDD (*n* = 14), six received concomitant IV antibiotic therapy for KPC-2-KP infection, (2) no SDD (*n* = 76), 22 received concomitant IV antibiotic therapy for KPC-2-KP infection	In-hospital mortality: 36 % vs. 45 %; decolonization (at day 21): 43 % vs. 17 %	Colistin: two patients receiving SDD (both also received colistin IV); gentamicin: five patients receiving SDD (three also received gentamicin IV)	“… SDD with gentamicin and colistin contributed to decolonization of KPC-2-KP in 6 of 14 cases (43 %) but revealed a substantial risk of rapid induction of secondary bacterial resistance to colistin and gentamicin.’

ESBL-KA, extended spectrum beta-lactamase-producing *Klebsiella aerogenes*; ESBL-KP, extended spectrum beta-lactamase-producing *Klebsiella pneumonia*; IV, intravenous antibiotics; KPC-2-KP, *Klebsiella pneumoniae* carbapenemase-2-producing *Klebsiella pneumonia*; LOS, length of stay; MDR, multidrug-resistant; MDR-E, multidrug-resistant Enterobacteriaceae; RCT, randomized controlled trial; SDD, selective digestive decontamination.

There are currently three RCTs evaluating the effects of SDD to decolonize patients that are carriers of MDR-GNB. However, these experimental studies, with 40 to 152 patients only, were all performed outside the ICU.

In a double-blind, placebo-controlled RCT in Switzerland, 58 hospitalized patients with intestinal carriage with ESBL-producing Enterobacteriaceae (in absence of infection) received either SDD (enteral colistin and neomycin, oral nitrofurantoine for 5 days in case of urinary tract carriage) or placebo [19]. Although SDD yielded an immediate decline in intestinal ESBL-producing Enterobacteriaceae carriage, treatment effects had disappeared 1 week after discontinuation of SDD.

In an Israeli hospital where CRE were endemic, the effect of targeted SDD on intestinal carriage with CRE was determined in a double-blind RCT [20]. Forty hospitalized patients with carbapenem-resistant *K. pneumonia* colonization or infection were randomized to 7 days of SDD (oropharyngeal and enteral gentamicin and colistin) or placebo. Decolonization rates in the intestinal tract after 1 week of SDD were 61.1 % with SDD and 16.1 % with placebo, but this difference declined at follow-up and was no longer significant after 5 weeks. Secondary resistance to gentamicin or colistin was not observed in any of the SDD-treated patients.

In an Israeli semi-RCT, patients with intestinal CRE carriage – mainly Klebsiella species – were treated with any of different SDD regimens (gentamicin for colistin-resistant CRE, colistin for gentamicin-resistant CRE and randomized allocation to colistin, gentamicin or both for CRE susceptible to both antibiotics), and eradication rates were compared with untreated patients (who did not consent to intervention or had CRE resistant to both gentamicin and colistin) [21]. Eradication of intestinal CRE carriage was achieved in 22 of 50 patients (44 %) treated with any SDD regimen and in seven of 102 untreated patients (7 %) (*P* <0.001). Secondary resistance developed in seven of the 50 SDD-treated patients (gentamicin resistance in six of 26 gentamicin-treated patients and colistin resistance in one of 16 colistin-treated patients).

## Conclusion

Based on studies performed in ICUs with low levels of antibiotic resistance – mainly from the Netherlands – there is no evidence that universal use of SDD or SOD increases antibiotic resistance among GNB, neither in individual patients or at ICU level. The evidence base for the effects of selective decontamination in ICUs where MDR-GNB are endemic is limited to observational data and one small RCT, all on SDD, yielding contradicting results. Targeted SDD for patients colonized with MDR-GNB has been studied in RCTs outside the ICU setting, where it seemed to result in short-term benefits only, with associated risks of resistance development to the antibiotics used. There is therefore currently insufficient evidence to recommend the use of SDD in settings with high levels of antibiotic resistance or to eradicate carriage with MDR-GNB. For the latter settings, more well-designed and sufficiently powered studies are needed. In ICUs with low levels of antibiotic resistance, SDD or SOD should be used only with careful microbiological monitoring for resistance development.

## Note

This article is part of a series on *Antibiotic resistance in the ICU*, edited by Steven Opal. Other articles in this series can be found at http://ccforum.com/series/antibioticresistance

## Abbreviations

CRE: carbapenem-resistant Enterobacteriaceae
ESBL: extended-spectrum beta-lactamase
GNB: Gram-negative bacteria
HRMO: highly resistant microorganisms
MDR-GNB: multidrug-resistant Gram-negative bacteria
MRSA: methicillin-resistant *Staphylococcus aureus*
RCT: randomized controlled trial
SDD: selective digestive decontamination
SOD: selective oropharyngeal decontamination
VRE: vancomycin-resistant enterococci

## References

[1] van der Waaij D, Berghuis-de Vries JM, der Lekkerkerk-van W (1972). Colonization resistance of the digestive tract and the spread of bacteria to the lymphatic organs in mice. J Hygiene.

[2] Price R, MacLennan G, Glen J (2014). Selective digestive or oropharyngeal decontamination and topical oropharyngeal chlorhexidine for prevention of death in general intensive care: systematic review and network meta-analysis. BMJ.

[3] Daneman N, Sarwar S, Fowler RA, Cuthbertson BH (2013). Effect of selective decontamination on antimicrobial resistance in intensive care units: a systematic review and meta-analysis. Lancet Infect Dis.

[4] Krueger WA, Lenhart FP, Neeser G, Ruckdeschel G, Schreckhase H, Eissner HJ (2002). Influence of combined intravenous and topical antibiotic prophylaxis on the incidence of infections, organ dysfunctions, and mortality in critically ill surgical patients: a prospective, stratified, randomized, double-blind, placebo-controlled clinical trial. Am J Respir Crit Care Med.

[5] de Jonge E, Schultz MJ, Spanjaard L, Bossuyt PM, Vroom MB, Dankert J (2003). Effects of selective decontamination of digestive tract on mortality and acquisition of resistant bacteria in intensive care: a randomised controlled trial. Lancet.

[6] Camus C, Bellissant E, Sebille V, Perrotin D, Garo B, Legras A (2005). Prevention of acquired infections in intubated patients with the combination of two decontamination regimens. Crit Care Med.

[7] de Smet AM, Kluytmans JA, Cooper BS, Mascini EM, Benus RF, van der Werf TS (2009). Decontamination of the digestive tract and oropharynx in ICU patients. N Engl J Med.

[8] de Smet AM, Kluytmans JA, Blok HE, Mascini EM, Benus RF, Bernards AT (2011). Selective digestive tract decontamination and selective oropharyngeal decontamination and antibiotic resistance in patients in intensive-care units: an open-label, clustered group-randomised, crossover study. Lancet Infect Dis.

[9] Oostdijk EA, Smits L, de Smet AM, Leverstein-van Hall MA, Kesecioglu J, Bonten MJ (2013). Colistin resistance in Gram-negative bacteria during prophylactic topical colistin use in intensive care units. Intensive Care Med.

[10] Oostdijk EA, de Smet AM, Kesecioglu J, Bonten MJ (2012). Decontamination of cephalosporin-resistant Enterobacteriaceae during selective digestive tract decontamination in intensive care units. J Antimicrob Chemother.

[11] Oostdijk EA, Kesecioglu J, Schultz MJ, Visser CE, de Jonge E, van Essen EH (2014). Effects of decontamination of the oropharynx and intestinal tract on antibiotic resistance in ICUs: a randomized clinical trial. JAMA.

[12] Oostdijk EA, de Smet AM, Blok HE, Thieme Groen ES, van Asselt GJ, Benus RF (2010). Ecological effects of selective decontamination on resistant Gram-negative bacterial colonization. Am J Respir Crit Care Med.

[13] Camus C, Salomon S, Bouchigny C, Gacouin A, Lavoue S, Donnio PY (2014). Short-term decline in all-cause acquired infections with the routine use of a decontamination regimen combining topical polymyxin, tobramycin, and amphotericin B with mupirocin and chlorhexidine in the ICU: a single-center experience. Crit Care Med.

[14] Houben AJ, Oostdijk EA, van der Voort PH, Monen JC, Bonten MJ, van der Bij AK (2014). Selective decontamination of the oropharynx and the digestive tract, and antimicrobial resistance: a 4 year ecological study in 38 intensive care units in the Netherlands. J Antimicrob Chemother.

[15] Heininger A, Meyer E, Schwab F, Marschal M, Unertl K, Krueger WA (2006). Effects of long-term routine use of selective digestive decontamination on antimicrobial resistance. Intensive Care Med.

[16] Ochoa-Ardila ME, Garcia-Canas A, Gomez-Mediavilla K, Gonzalez-Torralba A, Alia I, Garcia-Hierro P (2011). Long-term use of selective decontamination of the digestive tract does not increase antibiotic resistance: a 5-year prospective cohort study. Intensive Care Med.

[17] Wittekamp B, Oostdijk E, de Smet A, Bonten M (2015). Colistin and tobramycin resistance during long-term use of selective decontamination strategies in the intensive care unit: a post hoc analysis. Crit Care.

[18] Halaby T, Al Naiemi N, Kluytmans J, van der Palen J, Vandenbroucke-Grauls CM (2013). Emergence of colistin resistance in Enterobacteriaceae after the introduction of selective digestive tract decontamination in an intensive care unit. Antimicrob Agents Chemother.

[19] Huttner B, Haustein T, Uckay I, Renzi G, Stewardson A, Schaerrer D (2013). Decolonization of intestinal carriage of extended-spectrum beta-lactamase-producing Enterobacteriaceae with oral colistin and neomycin: a randomized, double-blind, placebo-controlled trial. J Antimicrob Chemother.

[20] Saidel-Odes L, Polachek H, Peled N, Riesenberg K, Schlaeffer F, Trabelsi Y (2012). A randomized, double-blind, placebo-controlled trial of selective digestive decontamination using oral gentamicin and oral polymyxin E for eradication of carbapenem-resistant Klebsiella pneumoniae carriage. Infect Control Hosp Epidemiol.

[21] Oren I, Sprecher H, Finkelstein R, Hadad S, Neuberger A, Hussein K (2013). Eradication of carbapenem-resistant Enterobacteriaceae gastrointestinal colonization with nonabsorbable oral antibiotic treatment: a prospective controlled trial. Am J Infect Control.

[22] Brun-Buisson C, Legrand P, Rauss A, Richard C, Montravers F, Besbes M (1989). Intestinal decontamination for control of nosocomial multiresistant gram-negative bacilli. Study of an outbreak in an intensive care unit. Ann Intern Med.

[23] Taylor ME, Oppenheim BA (1991). Selective decontamination of the gastrointestinal tract as an infection control measure. J Hosp Infect.

[24] Decré D, Gachot B, Lucet JC, Arlet G, Bergogne-Berezin E, Regnier B (1998). Clinical and bacteriologic epidemiology of extended-spectrum beta-lactamase-producing strains of Klebsiella pneumoniae in a medical intensive care unit. Clin Infect Dis.

[25] Agusti C, Pujol M, Argerich MJ, Ayats J, Badia M, Dominguez MA (2002). Short-term effect of the application of selective decontamination of the digestive tract on different body site reservoir ICU patients colonized by multi-resistant Acinetobacter baumannii. J Antimicrob Chemother.

[26] Lubbert C, Faucheux S, Becker-Rux D, Laudi S, Durrbeck A, Busch T (2013). Rapid emergence of secondary resistance to gentamicin and colistin following selective digestive decontamination in patients with KPC-2-producing Klebsiella pneumoniae: a single-centre experience. Int J Antimicrob Agents.

